# Osteocytes and Weightlessness

**DOI:** 10.1007/s11914-021-00713-8

**Published:** 2021-11-12

**Authors:** Donata Iandolo, Maura Strigini, Alain Guignandon, Laurence Vico

**Affiliations:** U1059 INSERM - SAINBIOSE (SAnté INgéniérie BIOlogie St-Etienne) Campus Santé Innovation, Université Jean Monnet, Saint-Priest-en-Jarez, France

**Keywords:** Osteocytes, Weightlessness, Bone cells, Cytokines, Lacuna-canalicular network, Peri-lacunar remodeling

## Abstract

**Purpose of Review:**

Osteocytes are considered to be the cells responsible for mastering the remodeling process that follows the exposure to unloading conditions. Given the invasiveness of bone biopsies in humans, both rodents and in vitro culture systems are largely adopted as models for studies in space missions or in simulated microgravity conditions models on Earth.

**Recent Findings:**

After a brief recall of the main changes in bone mass and osteoclastic and osteoblastic activities in space-related models, this review focuses on the potential role of osteocytes in directing these changes. The role of the best-known signalling molecules is questioned, in particular in relation to osteocyte apoptosis.

**Summary:**

The mechanotransduction actors identified in spatial conditions and the problems related to fluid flow and shear stress changes, probably enhanced by the alteration in fluid flow and lack of convection during spaceflight, are recalled and discussed.

## Introduction

The International Space Station (ISS) is in perpetual free fall above the Earth. Its propulsion speed is equal to the speed of its “fall” toward the Earth. Thus, the astronauts and all the objects inside float, experiencing so-called microgravity. Important for this review, beyond “weightlessness,” microgravity also induces lack of hydrostatic pressure, of buoyancy, and convection movements [1]. The ISS and unmanned spacecrafts are unique international facilities for life sciences where research can be conducted on whole organisms and cultured cell. As space missions are rare, space analogues on Earth have been developed such as bed rest and dry immersion for humans, hindlimb unloading (HU) for rodents, and clinostats (e.g., random positioning machines) for cell culture. Long-duration bed rest is the most popular model in humans to simulate the effects of microgravity on various physiological systems, especially for studies on bone, muscle, and the cardiovascular system [2]. In the dry immersion model, the subject is immersed in water in a supine position. Unlike bedrest, it does not contemplate mechanical support for the body and it leads to effects that are more severe and/or appearing at earlier times than in support-loaded bed rest [3]. Still, in order to more directly investigate the cellular and molecular mechanisms of bone adaptation to microgravity, invasive tools are necessary to run more informative onsite analysis of those cells whose metabolism is known to be site-dependent (e.g., osteoblasts, osteocytes, and osteoclasts). After a brief recall of unbalanced bone remodeling activities and consequent bone loss in space (“Bone Loss and Increased Bone Resorption in Space-Related Conditions” section), this review concentrates on osteocytes, the key regulators of bone homeostasis and skeletal mechano-sensation and transduction during unloading.

Mechanical load (or its absence) eventually leads to the modulation of the expression and release from osteocytes of several secreted signalling molecules that, crucially, target other bone cells and influence their activity; these include SOST (“Sclerostin: the Cause of Decreased Osteoblastic Bone Formation in Weightlessness?” section) and RANKL (“Osteocyte RANKL: the Cause of Osteoclastic Bone Resorption?” section) [4].

A number of cellular structures have been shown to play a role in the mechanotransduction within the lacuno-canalicular space (LCS). Osteocytic mechanosensory system, cytoskeleton, primary cilia, connexin-hemichannels, ion channels (Piezo1), or membrane receptors such as parathyroid hormone receptor type 1 (PTH1R) ligands could all be affected in microgravity-related conditions [5, 6]. The identity of the receptor molecules and the signal transduction pathways triggered downstream of mechano-receptor activation have been detailed in two recent reviews [7, 8]. We will report some recent updates in “Recent Updates on the Responses of Osteocytes to Weightlessness at the Cellular and Molecular Level” section. The potential role of osteocyte apoptosis or senescence in the intrinsic and complex mechanism underlying bone loss during unloading remains ill-defined (“Is Space-Flight Bone Loss Linked to Osteocyte Apoptosis or Senescence?” section). Osteocytes sense and respond to mechanical stimuli, by detecting substrate stretching or the shear stress generated by the fluid flow. As microgravity-related conditions are associated to major fluid perturbation, osteocytes’ functions (e.g., mechanosensing, control of matrix-mineralization, and bone remodeling) may be dramatically affected (“Changes in Osteocyte Connectivity and LCN Flow” section).

Overall, this review discusses how dysregulation of all these osteocyte functions may contribute to the rapid decrease of bone in space-related conditions, pointing to unanswered questions mainly associated to the microgravity-related loss of connectivity in the lacuno-canalicular system.

## Bone Loss and Increased Bone Resorption in Space-Related Conditions

In space, despite the measures implemented to protect the musculoskeletal system of crew members, such as strictly planned physical activities and diets, significant bone loss occurs mainly at weight-bearing skeletal sites [9]. At these locations, bone microstructural changes were found to affect both the trabecular and cortical compartments as assessed by high-resolution peripheral quantitative computed tomography (HRpQCT).

The cellular response is predominantly characterized by increased bone resorption, a very robust trait, while more variable observations are reported for bone formation. Changes in the serum levels of resorption markers after 4.5- to 6-month spaceflights were found negatively associated with those of tibia trabecular number and positively associated with those of tibia cortical porosity [10]. These relationships highlight the importance of bone resorption in reshaping both trabecular and cortical structures. In a meta-analysis of bone loss in space travelers, Stavnichuk and colleagues estimated that bone resorption markers increased hyperbolically with a time to half-max of 11 days and plateaued at 113% above pre-flight levels, while bone formation markers remained unchanged during the first 30 days and increased thereafter at 7% per month, as a consequence of bone turnover coupling [11]. Studies in space analogue models revealed that bone resorption markers were already increased on the second day of bed rest [12, 13], and as early as the first day in the dry-immersion model [3].

The cause of the changes in resorption and formation activity is thought to be related, to a large part, to changes concerning osteocytes. Located deep within the bone matrix, but exquisitely mechanosensitive thanks to their morphology and interconnected network, osteocytes may act on surface bone cells (osteoblasts and osteoclasts) by many different pathways. The most popular are the production of the cytokines sclerostin (encoded by the gene SOST) and receptor activator of nuclear factor κB ligand (RANKL), being regulated by mechanical cues [4]. Sclerostin is an inhibitor of bone formation, and mice with osteocytes not producing it, do not undergo bone loss when they are submitted to HU [14]. Similarly, mice lacking osteocyte RANKL, a major regulator of osteoclastogenesis, show no bone loss during unloading [15]. The osteocytic cell line Ocy454 when cultured in the NASA rotating wall vessel system, an in vitro microgravity simulator, displayed increased expression of both sclerostin and RANKL [16]. The fact that these in vitro results reproduce the in vivo observations suggests that sclerostin and RANKL upregulation can be direct, cell-autonomous effects of unloading on osteocytes.

## Sclerostin: the Cause of Decreased Osteoblastic Bone Formation in Weightlessness?

Trends toward increased serum sclerostin levels were found in both astronauts [17] and cosmonauts [10] after 4- to 6-month sojourns in the International Space Station (ISS). Sclerostin levels increased by about 10% at day 11 and 14 in two bed rest studies of 14 and 21 days, respectively, and they declined at the end of the bed rest periods [18]. In a longer bed rest trial (90 days), sclerostin was found to be increased by 29% at day 28, 42% at day 60, and only by 22% at day 90 with great interindividual variations [19]. In a 3-day dry immersion model, no change was seen, suggesting that sclerostin is not an early responder [3]. Thus, in humans, microgravity and microgravity analogues produce either increase or no change in serum sclerostin level, in line with bone formation markers. Because of both technical and ethical limitations, it is not known to what extent sclerostin levels change locally in bones under these conditions. This could instead be directly analyzed in animal models. The effects of spaceflight on SOST expression have been studied in mice calvaria after the 30-day BION M1 mission. Despite no change in the calvaria structure, SOST mRNA expression was 16-fold greater in the spaceflight group than that in the ground control group [20]. The percentage of mandibular sclerostin-positive osteocytes was also found greatest in mice both after the 15-day STS-135 and the 30-day BION-M1 space missions [21]. Surprisingly, to the best of our knowledge, no data on SOST expression in the long bones of mice subjected to space flight are available in literature. Regenerating goldfish scales incubated during 86 h in the ISS under microgravity conditions demonstrated increased SOST expression in both osteoblast and osteocyte-like cells [22]. Sclerostin-positive osteocytes were found increased in the cortex of the proximal tibial metaphysis of 2-week HU mice [23]. In a recent paper, Osumi and colleagues demonstrated that in rats subjected to mechanical unloading of hindlimbs by sciatic nerve dissection, unloading induced a redistribution of sclerostin staining in the distal femur metaphysis where bone loss was observed [24••]. Here, the most marked increase in the average intensity of sclerostin distribution appeared at the level of the periosteal side in the lateral regions. The authors adopted transmission immuno-electron microscopy to attain high-resolution evaluation of the distribution of sclerostin in this area. First, the concentration of sclerostin outside of the osteocyte cell body appeared to be higher than in cell body or lacunae. Second, sclerostin density in the canaliculi was found to be significantly higher in the unloading group than in the sham group, but unchanged in the lacunae and cell body. They suggested that, in response to mechanical unloading, it is changes in canalicular solute transport (see also “Changes in Osteocyte Connectivity and LCN Flow” section) that affect the distribution of sclerostin in the impacted bone areas, rather than changes in sclerostin synthesis.

Periostin is an extracellular protein expressed by osteoblasts, in particular in the periosteum. It works as a positive regulator of bone anabolism by decreasing SOST expression [25]. Gerbaix and co-authors showed that HU in mice inhibits periostin expression in the femur cortical bone, which then induces decreases in cortical bone volume and thickness at the femoral midshaft associated with an increase in SOST [26]. These results further indicate that periostin decrease with the consecutive sclerostin increase is an essential mediator of cortical bone response not only to loading but also to unloading. In cosmonauts, although periostin serum level was not significantly altered after 4.5- to 6-month spaceflight, its serum level was showed to predict tibia cortical evolution during space mission, their respective pre-post flight changes being negatively correlated at landing [10]. In the same missions, periostin was found to predict tibia cortical thickness and BMD recovery after landing [10].

## Osteocyte RANKL: the Cause of Osteoclastic Bone Resorption?

In mice as in humans, bone loss is associated to bone resorption causing a thinning and disconnection of trabeculae, as seen after the one-month BION M1 involving male C57BL/6 mature mice [27••]. In this mission, synchrotron analysis on about 6000 osteocytic lacunae in the femur diaphysis revealed that lacunar volume is reduced displaying a more spherical shape. Such osteocyte lacuna rounder shape was also observed after the 91-day ISS mission (called Mice Drawer System mission) in the sole surviving wild-type mouse [28]. In the BION M1 mission, through classical histology, we observed that the number of empty lacunae is more than tripled [27••], suggesting massive osteocyte death. In another study, female C57BL/6 mature mice included in the 14-day STS-131 mission displayed increased osteocyte lacunar volume in the ischium with osteocytes expressing more stromelysin-2 (MMP10), supporting a role for osteocytic osteolysis in the bone loss of these animals [29]. Whether the difference in these results is related to sex, bone site, and/or spaceflight duration requires further investigation.

As in actual spaceflight, in skeletally mature animals, bone adapts to HU with features typical of disuse-induced bone loss, mostly explained as a consequence of an increased resorption activity rather than a decreased formation activity [30–33]. Higher resorption is primarily dependent on elevated RANKL signaling. In HU experiments with mice in which RANKL is specifically knocked-out in osteocytes (obtained with the Dmp1-Cre), Xiong and colleagues showed that osteocyte-derived RANKL is responsible, to a large extent, for increased osteoclast numbers and both cancellous and cortical bone loss due to mechanical unloading [15].

Osteocyte apoptosis has been proposed as the cause of osteoclastic activation during unloading, and several teams have reported results that might support this mechanistic explanation [30, 33–37] (see also “Is Space-Flight Bone Loss Linked to Osteocyte Apoptosis or Senescence?” section below). However, only the study by Cabahug-Zuckerman and colleagues concluded that, in HU, osteocyte apoptosis plays a regulatory role in triggering RANKL production by osteocytes with the consequent activation of new resorption and therefore bone loss [30]. The model was based on the observation that RANKL upregulation and bone loss were blunted in mechanically unloaded mice treated with the pan-caspase apoptosis inhibitor QVD (quinolyl-valyl-O-methylaspartyl-[-2,6-difluorophenoxy]-methylketone). Although Cabahug-Zuckerman and collaborators could exclude a direct effect of QVD on RANKL gene expression in cultured osteocytes and osteoclastogenesis in vitro, it should be kept in mind that this treatment does not specifically inhibit apoptosis only in osteocytes. On the contrary, Plotkin and colleagues used a bisphosphonate analogue (IG9402) to specifically reduce osteocyte apoptosis. The treatment prevented the increase in osteocytic RANKL expression, but failed to inhibit resorption or bone loss in HU female 4-month-old C57BL/6 mice, while not affecting osteoblast apoptosis and its RANKL expression [33]. We are not necessarily able to reconcile these two studies, but we note that the major difference is that in the work by Cabahug-Zuckerman and colleagues the adopted pan-caspase apoptosis inhibitor would affect all cells indiscriminately, whereas in the study by Plotkin and collaborators, only osteoblasts and osteocytes were affected. It is possible that RANKL from osteoblasts might be sufficient to induce osteoclastogenesis and the loss of bone mass during unloading.

Moreover, recently a new source of RANKL has been discovered. Two studies have provided evidence for a critical role of bone marrow adipocyte-derived RANKL in osteoclastogenesis and bone remodeling [38, 39]. Both models of bone loss displayed increased marrow fat and excessive osteoclastogenesis and bone resorption accompanied by RANKL in the trabecular compartment. The two studies used adipoq-Cre to specifically target adipocytes to show the functional relevance of RANKL secreted by bone marrow adipocytes. However, Onji and colleagues warn that in Adipoq-Cre reporter mice, a few osteocytes and osteoblasts are Cre-positive in aged mice [39, 40]. Adiponectin (Adipoq), used as a marker of mature and pre-adipocytes, was also seen in osteoblasts [41]. Therefore, Adipoq-Cre knock-out mice could also target RANKL expression in osteoblasts, and osteocytes.

In brief, RANKL is expressed by a variety of cell types, including chondrocytes, lymphocytes, adipocytes, osteoblasts, and osteocytes and it is still unclear which of them are essential RANKL sources to support osteoclast formation. It could well be that osteocyte-derived RANKL regulates cortical bone homeostasis, while adipocyte lineage and osteoblast-derived RANKL is in charge of trabecular bone homeostasis. It would be important to perform additional experiments—including unloading-induced bone loss—using different constitutive or inducible Cre lines to corroborate this hypothesis.

One should also keep in mind that the ratio RANKL/osteoprotegerin (OPG) is the important factor to consider. OPG is a soluble decoy receptor that binds RANKL preventing its binding to RANK, thereby limiting bone resorption. Thus, it is quite possible that increased bone resorption under microgravity conditions can also be the result of a reduced OPG production, rather than of an increased RANKL production. This hypothesis was indeed demonstrated by mice treated with excess glucocorticoid. Here, the changes in endocortical resorption were driven by reduced OPG (produced by osteoblasts and osteocytes, B-cells and dendritic cells) and not by elevated RANKL levels [42].

## Recent Updates on the Responses of Osteocytes to Weightlessness at the Cellular and Molecular Level

Early events in mechano-transduction in osteocytes have not been directly studied in microgravity conditions and extrapolations of what may happen in space are based on in vitro studies. For more detailed reviews on osteocyte mechanotransduction and downstream cellular and molecular events, we refer the reader to recent reviews [7, 8], and the other contributions to this special issue on osteocytes. Recently, it has been possible to more comprehensively study the molecular and cellular responses of osteocytes in actual spaceflight situations, primarily thanks to molecular/transcriptomic analyses of cultured cells and in animals exposed to spaceflight, allowing for a more accurate comparison between simulated and actual microgravity conditions. We report below on recent literature specific to cellular and molecular responses of osteocytes to real or simulated weightlessness.

### Glucose and Energy Metabolism

In a recent article, Uda and colleagues perform transcriptomics on osteocyte cultures obtained from the Ocy454 late osteoblast-early osteocyte cell line, kept for up to 6 days in static culture aboard the SpaceX Dragon rocket during SpaceX Dragon-6 resupply mission to the International Space Station [43••]. Results were compared to those of a transcriptomic analysis of Ocy454 cells exposed to microgravity-simulating conditions on the ground (rotating wall vessels), and to those on other cell types from NASA databases. Interestingly, genes involved in glucose metabolism and ATP consumption are the most affected by spaceflight microgravity, reflecting an increase in glucose metabolism and oxygen consumption, while no change in the expression of genes involved in apoptosis and senescence was detected between ground and space conditions. Of note, results from Ocy454 cells cultured for the same study under simulated microgravity do not align with those from spaceflight, pointing to an inability of ground models to mimic spaceflight. Whether this is an inherent limitation of ground models or a shortcoming of the specific one used in this study is debatable.

Under diamagnetic levitation as a novel ground-based model for simulating a reduced gravity environment, Wang and colleagues observed lower expression of energy metabolism related genes in osteocyte in simulated microgravity-exposed MLO-Y4 [44]. Even if metabolism of osteocytes is highly dependent on culture conditions (CO_2_-independent medium, hypoxia), further metabolomic studies could contribute significantly to the understanding of gravity-induced integrative effects in osteocytes.

In space, we know that the cytoskeleton of bone-forming cells is destabilized [45]. Post-translational modifications (PTMs) of cytoskeletal structures that increase tubulin stability include acetylation and glutamylation [46]. Therefore, these PTMs rely on the availability of acetylCoA and glutamine. It was recently shown that in cancer cells pharmacologic inhibition of glutamine metabolism decreased microtubule (MT) glutamylation, affecting their mechanical stabilization [47]. Whether initial metabolic stress impacts cytoskeleton stability or whether the need of MT stability diverts cell metabolites remains to be established. We may hypothesize that osteocytes exposed to microgravity-related conditions use a part of the acetyl-CoA or glutamine/glutamate pool to stabilize their MT, thus diverting them from their physiological metabolic routes. All together, these data may help to understand why so many metabolic and mitochondrial markers are affected by microgravity. Given the extraordinary complexity of the osteocyte cytoskeletal network within the lacuno-canalicular network (LCN)/lacuno-canalicular system (LCS), it is easy to imagine that maintenance of its stability is a metabolic challenge.

#### Primary Cilia

Primary cilia are essential components of the mechanosensing apparatus in osteocytes. Ding and colleagues recently showed that osteocyte cilia are shorter in MLO-Y4 osteocyte-like cells cultivated for 2 days in simulated microgravity condition (using a device rotating at 15 revolutions per min, such that the sum of the gravity force vector is zero) [48]. Cilia shortening is accompanied by, and is suggested to be a consequence of, a reduction in the size of anterogradely moving intra-flagellar transport particles and altered motor-protein dependent movement of particles in cilia. Therefore, the sensing capability of osteocytes in microgravity may be diminished, possibly further decreasing the response to already less intense mechanical stimuli. While the study of intra-flagellar transport in vivo on osteocytes can be tricky, it is tempting to propose to measure primary cilia of osteocytes via acetylated-tubulin staining [49] in bone sections to confirm a potential shortening in microgravity.

#### Piezo1

Piezo1 is a member of the newly discovered Piezo family of mechano-sensitive ion channels. The loss of Piezo1 from the osteoblastic lineage (osteoblasts and osteocytes) was proved to lead to decreased bone density, increased bone resorption and to more frequent fractures [50••, 51••]. Subjecting osteoblasts to microgravity simulation in vitro by culturing them in the RPM induces a decrease in osteoblast Piezo1 expression [50••]. In addition, and relevant to space-flight bone biology, conditional deletion of Piezo1 in both osteoblasts and osteocytes (with the Prx1-Cre or the Ocn-Cre lines) prevents the further loss of bone in response to HU [50••, 51••], while loss of Piezo in osteoclasts (obtained with the Ctsk-Cre line) does not [51••]. To what extent loss of Piezo specifically in osteocytes only (as opposed to osteoblasts) would also prevent HU-induced bone loss has not been tested, nor have these mice been sent to space yet. The expression of many genes is altered in mutant Piezo1 bones, though apparently not that of the “usual suspects” key regulators of bone homeostasis, such as MCSF, RANKL, OPG, SOST, Semaphorin 3. Rather, collagen genes are affected [51••].

In addition to Piezo1, the YAP and TAZ proteins, transcriptional coactivators encoded by paralogous genes, which shuttle between the cytoplasm and the nucleus in response to multiple inputs, have since become of wide interest in many fields, including mechanobiology [52, 53]. In vitro, Piezo1 is required to upregulate Collagen 2 and Collagen 9 expression in preosteoblasts upon mechanical stimulation, possibly in a YAP-dependent manner, and over-expression of these two collagen genes appears to blunt pro-osteoclastogenic activity of osteoblast [51••]. Although extensively studied on earth, this target should be explored in the near future in the field of microgravity. Importantly, YAP and TAZ mediate osteocyte perilacunar/canalicular remodeling (PLR) [54], as discussed later on in “Changes in Osteocyte Connectivity and LCN Flow” section.

#### Leukemia Inhibitory Factor

Leukemia inhibitory factor (LIF) is a member of the interleukin-6 family of cytokines with highly pleiotropic biological activities. LIF expression has been reported for all three main bone cells by different groups [55]. Recently, LIF was shown to be released into the blood at high levels of mechanical stimulation; conversely, its bone and serum levels decrease in tail-suspended mice, as well as in MLO-Y4 cells grown under random positioning machine (RPM) [55]. Khosla’s group identified LIF as one of the factors present in both serum and bone whose levels decrease in post-menopausal women. Of note, transcriptomic analysis on post-menopausal women identified LIF as a putative osteoclast-produced coupling factor [56].

It remains to be seen if LIF levels are modulated by actual spaceflight in bone and to what extent osteoclast-derived and/or osteocyte-derived pools are affected.

## Is Space-Flight Bone Loss Linked to Osteocyte Apoptosis or Senescence?

In various physiological and pathological situations, cells in a tissue may undergo apoptosis, a form of programmed cell death, or enter a death-resistant, non-proliferative and highly secretory state called senescence. Apoptosis and, more recently, senescence of bone cells, in particular of the osteoblast-osteocyte lineages, are considered as important causes of bone loss.

Apoptosis has been reported to occur in several osteoporosis models, including sex hormone deprivation (ovariectomy, orchiectomy), aging, and glucorticoid-induced osteoporosis [8, 57, 58]. Specifically for microgravity models, TUNEL staining, immunolabeling of apoptosis effector proteins Caspase 3 and 9 or pro-apoptotic markers like Bax and analysis of the morphology and organization of the osteocytic LCN showed osteoblast and osteocyte apoptosis in mice and rats returning from the ISS [27••] or subjected to mechanical unloading on Earth [30, 33, 34] (but discording results have also been reported [59]). Molecular biology and biochemical analysis of apoptotic markers support those observations in many instances. Osteocytic apoptosis correlates with and, crucially, precedes increased RANKL and SOST expression (at least in most cases analyzed). Transcript and protein levels are usually measured in extracts from cortical bone, highly enriched in osteocytes, or visualized by immunostaining on bone sections. The increased secretion of these factors, capable to modulate bone homeostasis, occurs in osteocytes adjacent to dying osteocytes, particularly clear in fatigue-induced microdamage where osteocyte death is not uniform within the cortex [60]. Data from in vivo [61], and in vitro micro-fluidics-assisted complex co-culture systems [62] show that ATP released by apoptotic osteocytes stimulates RANKL expression in nearby healthy osteocytes, in a Pannexin-1 and P2X7 ATP-receptor dependent manner. This has been reported in an in vivo model of fatigue-linked bone loss, and not yet in actual or simulated microgravity models, while in the in vitro study osteocyte apoptosis was induced by heat shock. Most importantly, inhibition of apoptosis by chemical [30, 33] or genetic approaches indirectly affecting apoptosis of bone cells [63] provided functional evidence for a causal link between apoptotic programmed cell death and bone loss, rather than mere co-occurrence. Transgenic mice over-expressing the gap junction component Connexin43 show fewer apoptotic osteocytes and preserved bone homeostasis in aged mice [63]. Loss of Connexin43 in osteocytes leads to more apoptosis and bone loss at baseline, but also to protection from further bone loss upon HU [64, 65].

A recent paper revealed that the ability of the myokine irisin to prevent disuse-induced bone loss in tail suspended mice is linked to its anti-apoptotic action on bone cells [66]. This may suggest that osteocytic apoptosis contributes to HU-induced bone loss. Irisin appears to target other bone cell types and affect their viability/differentiation or rescue them from programmed cell death [67–71]. Chen and collaborators showed a positive effect of irisin on the differentiation of osteoblasts in simulated microgravity models in vivo and in vitro; this would occur by increasing beta-catenin levels and thus possibly Wnt signalling [72]. So, as with other approaches that interfere with osteocyte apoptosis, not enough specificity has been achieved to confidently attribute a prime role for osteocyte apoptosis in unloading-dependent bone loss.

RT-PCR analysis shows that MLO-Y4 subjected to simulated microgravity express higher levels of the pro-apoptotic marker Bax and Bad [55], providing some, though by far not conclusive, evidence that unloading induced apoptosis occurs in vitro under simulated microgravity conditions. Obviously, it is hard to unequivocally attribute programmed cell death to microgravity as opposed to sub-optimal culture conditions. Once such doubts cleared, and with appropriate pure or co-culture conditions, in vitro models represent more amenable systems where to study the molecular mechanisms of apoptosis induction by weightlessness and its consequences. The transcriptomic analysis by the Divieti-Pajevic laboratory on Ocy454 exposed in vitro to weightlessness for up to 6 days did not show a clear apoptosis signature, though it identified a couple of pro-apoptotic genes (Egln3 and Fam162a) among those upregulated by microgravity [43••]. It is thus not clear whether osteocytes would enter apoptosis in a cell autonomous manner in microgravity, or as a response to local or systemic signals.

Senescence is not only observed but it appears to be causally linked with aging-related osteoporosis [73]. Several markers, p16Ink4a mRNA, EGFP mRNA encoded by the INK-ATTAC transgene (reporting p16Ink4a expression), and senescence-associated distension of satellites (SADS)-positive signal, are clearly detected in osteoporotic bone of aged mice, in particular in osteocytes [73, 74]. Genetic or pharmacological elimination of senescent cells, though admittedly not specifically of senescent osteocytes, prevented age-related bone loss in mice. On the contrary, senescence is not detected in, nor causally linked to, osteoporosis induced by sex-hormone deprivation [75]. No dedicated analysis of the senescent phenotype of osteocytes from HU or space-flight samples has been published to date. Blaber and co-workers did report an increase in p21 (one of the many markers of senescence/cell cycle arrest), but in osteoblasts rather than osteocytes [29]. Obviously, their analysis could not have been exhaustive because it predates the recently detailed molecular fingerprint of the senescent phenotype. In their extensive transcriptomic study of cultured Ocy454 subjected to microgravity for up to 6 days study, Uda and collaborators specifically comment that their analysis did not reveal any sign of senescence (nor autophagy) [43••].

In the years to come, studies on the occurrence of apoptosis and senescence in osteocytes subjected to microgravity, and their functional relevance to the bone loss observed in space-flight, will profit from the combined exploitation of in vivo and in vitro systems, both in real and simulated microgravity, including bone organoids. Their culture is said to be readily adaptable for space-flight modules and would combine the complexity and physiological relevance of multiple cell type organ-like structures with the accessibility of in vitro systems [76].

## Changes in Osteocyte Connectivity and LCN Flow

Measuring fluid flow into LCN has always been challenging and this type of measurement [77, 78] has never been done in microgravity. Recent numerical simulations have provided valuable information in an attempt to explain how the decrease in liquid movement under microgravity (i.e., decreased LCN flow) can cause osteoporosis in astronauts [79••]. For example, the flow field in an osteon as well as the mechanical response of osteocytes to the flow field under different gravity conditions were studied [80••]. The authors suggested that in the tested conditions of microgravity, osteocytes experience reduced liquid flow velocity in the LCN and fluid shear stress, therefore receiving less nutrients and insufficient mechanical stimulation.

We suggest that under reduced fluid flow, RANKL might not be responsible for the increase in resorption, as its diffusion to osteoclasts would be limited. In addition, because of limited diffusion, OPG might not be able to block residual RANKL, thus allowing osteoclastogenesis. Similarly, the diffusion of prostaglandin 2 (PGE2), ATP, and nitric oxide (NO) (all positive regulators of osteoblast function) [81] and Sclerostin might be hindered, which may interfere with osteoblastogenesis. In the future, studies to validate this hypothesis will be needed.

In space, in order to reach their target cells, these molecules might pass through the pericellular matrix (PCM) in the LCN only by diffusion thus requiring a continuous pericanalicular remodeling (PLR) to keep the LCN functional [82, 83]. On Earth, physiological levels of flow in the LCS, mechanical environment, and osteocyte mechanotransduction machinery (including YAP/TAZ) control PLR [53, 84]. In unloading conditions, it is tempting to speculate that PLR is not initiated or delayed thus explaining osteocyte deconditioning in space. Whether osteocytes are initiating PCM remodeling or bone osteolysis or both in unloading conditions need further investigations. Even if the physiological context is quite different, lactation (associated with reversible PLR) may provide interesting cues for understanding osteocyte response to unloading or disuse challenges. Recently, Lai and collaborators hypothesized that lactation-induced LCS changes in the maternal skeleton increase the fluid flow-mediated mechanical stimulation of osteocytes and transport of signaling molecules in the LCN, which may contribute to its rapid recovery after lactation [85]. Signals driving the rapid recovery post-lactation, however, remain unclear, although altered fluid/solute transport and osteocyte mechanosensing were proposed to be involved. Following on from the suggested link between lactation and recovery post-spaceflight, we could suggest that the same factors driving the recovery after lactation might be targets of interest to achieve better recovery after spaceflight.

PLR is a complex process that requires appropriate techniques to be studied especially in the field of gravitational physiology. If X-ray computed tomographies are suitable for mineralized tissue examination, highest resolution imaging techniques are appropriate to appraise osteocytic osteolysis but not for PCM examination or the evaluation of osteocyte architecture reorganization. Sarah Dallas and her team developed multiplexed confocal imaging methods for visualizing intact osteocytes in situ within bone tissue, using fixed whole mount neonatal mouse bones or thick frozen sections of adult mouse femur [86]. These methods allowed to image the osteocyte cell membrane, nucleus, cytoskeleton, and the LCS together with bone extracellular matrix proteins in various combinations. The main limitation of confocal imaging is the Z resolution. However, with the development of new protocols for bone tissue clearing and live cell imaging using super-resolution optical microscopy, accurate analysis of osteocytes’ dendrites and canaliculi can be performed [87]. These new technologies will lead to further insights into the ultrastructural biology of these cells and can be of high interest for exploration of space effects on osteocytes.

Due to limitation of flown bone samples to study osteocytes in vivo, osteocytic cell lines (e.g., MLO-Y4, -A5, Ocy454, IDGSW-3) can be of interest to decipher specific molecular regulation or signaling pathways as well as for studying the transition from osteoblast to mature osteocyte. Nevertheless, as changes of the LCS represent a biological signature of the mechanotransduction activity in response to external biomechanical loading, a 3D environment closely mimicking native bone should be provided for future in vitro experiments in gravitational physiology [88]. MLO-Y4 seeded in collagen-hydroxyapatite gel and cultured in a rotating wall vessel demonstrated that 3D conditions downregulate osteocytic markers and potentially desensitize them to mechanical cues [89]. Clinorotation downregulated DMP1 and E11 gene, and these experiments emphasized the complexity of studying osteocyte cell lines in a relevant environment.

The OmGFP66 clone generated by the team lead by Sarah Dallas showed a complete osteocyte gene expression profile, while forming organized bone nodule-like mineral, as osteocytes do in their natural microenvironment [90]. This cell line can be of great interest in future in vitro experiments in space.

In line with the need of native bone environment, bone organoids represent interesting and innovative tools, as proposed by Iordaschescu and collaborators, trabecular organoids are suitable for the study the effects of microgravity and degeneration [76]. Organoids and organotypic cultures are becoming an increasingly complex platform for replicating anatomical structures similar to human organs, they are particularly adapted for space research due to their reduced size, allowing for numerous permutations of subjected conditions.

## Conclusions and Recommendations

Bone loss is not a specific problem related to space sojourns and similarities with unloading and aging on Earth have been noticed long ago. However, the rapid bone loss (about 10 times faster than an elderly subject on Earth) shows that space is a unique and extreme model. Moreover, the bone modifications, more significant in the weight-bearing zones, point to local defects in the organization of the osteocyte network. In opposition to fibroblast growth factor 23 (FGF23), which acts as a hormone, sclerostin, OPG, and RANKL act more locally and their sampling should be more relevant in bone tissue/section than in blood. As laser capture bone microdissection is more accessible to the community [91], we may have means to better select areas rich in osteocytes. Single-cell RNA sequencing (scRNA-Seq) is also emerging as a powerful technology to examine transcriptomes of individual osteoblasts [92] and could be of interest for osteocytes. PLR is emerging as a quality control system necessary to ensure and maintain osteocyte functions. How microgravity is altering PLR may provide new findings on osteocyte mechanosensing and senescence activation. In addition to single cell activity, fluid flow and shear strain rate cannot be easily measured experimentally at the level of osteocyte and canaliculi within the bone matrix. Future studies should address the relation between mechanical cues and cellular activity using 3D image analysis along with computational fluid dynamics. Rodent skeletal tissue and robust perfused 3D in vitro models are required to accommodate high resolution of the finite elements to account for the lacuno-canalicular network. As compared to unloading situations on Earth, weightlessness-related interstitial fluidic alteration and lack of convection will exaggerate the decrease in nutrient/waste transport, metabolite trafficking, and shear stress. Such investigations seem particularly relevant in a situation of lack of G vector guidance to orient extracellular bone components and bony microstructures. How osteocytes’ functions are restored or delayed after Earth return is also of interest since the bone lost after several months in space does not seem to be fully recoverable [10].

## Data Availability

Not applicable.
